# Bilateral Temporomandibular Joint Changes Following Unilateral Posterior Occlusal Alteration in Rats: A Longitudinal MRI and Histomorphometric Study

**DOI:** 10.3390/dj14100639

**Published:** 2026-10-04

**Authors:** Silvia del Cueto, Angel Alvarez-Arenal, Serafín Costilla, Mirian Teulé-Trull, José A. Vega, José Martín-Cruces

**Affiliations:** 1 Departamento de Morfología y Biología Celular, Universidad de Oviedo, 33006 Oviedo, Spain; 2 Departamento de Cirugía y Especialidades Médico-Quirúrgicas, Área de Estomatología, Universidad de Oviedo, 33006 Oviedo, Spain; arenal@uniovi.es; 3 Departamento de Medicina, Área de Radiología, Universidad de Oviedo, 33006 Oviedo, Spain; costillaserafin@uniovi.es; 4 Master ENDORE, Universidad de Santiago de Compostela, 15705 Santiago de Compostela, Spain; miriam.teule.trull@usc.es (M.T.-T.); jose.martin.cruces@usc.es (J.M.-C.); 5 Facultad de Ciencias de la Salud, Universidad Autónoma de Chile, Santiago 750012, Chile

**Keywords:** articular disc, histology, magnetic resonance imaging, occlusion, rat, temporomandibular joint

## Abstract

**Background/Objectives**: Whether a unilateral posterior occlusal alteration produces bilateral temporomandibular joint (TMJ) changes, and how imaging findings relate to tissue-level alterations, remains incompletely characterised. This study aimed to characterise the bilateral TMJ response to a sustained unilateral posterior occlusal alteration in rats using longitudinal magnetic resonance imaging (MRI) and endpoint histological and histomorphometric assessment. **Methods**: Twenty-eight female Wistar rats were allocated to an experimental group (n = 12), a baseline control group (n = 4) and an age-matched control group (n = 12). Light-cured composite resin was applied to the three right maxillary molars of experimental animals and maintained for 16 weeks. Baseline MRI was obtained from the separate 8-week control group, whereas experimental and age-matched control animals underwent bilateral MRI at 16 and 24 weeks of age. Cartilage and disc structure, thickness and cell density were assessed histologically at the endpoint. **Results**: MRI demonstrated a progressive bilateral response. At 24 weeks, 11 of 12 experimental animals showed bilateral joint-space narrowing; condylar flattening affected 9 of 12 TMJs on each side, and reduced disc size was present in all experimental TMJs. Histology and histomorphometry showed a more intense ipsilateral response, with cartilage and disc thickening, loss of normal cartilage organisation and reduced cell density. Contralateral tissue architecture remained preserved, although disc thickness was greater than in age-matched controls. **Conclusions**: Unilateral posterior occlusal alteration produced a bilateral but asymmetric TMJ response: imaging abnormalities progressed in both joints, while tissue remodelling was most pronounced ipsilaterally. The combined findings support early load-related degenerative remodelling and show that unilateral occlusal disturbance can affect both TMJs to different degrees.

## 1. Introduction

The temporomandibular joint (TMJ) is a specialised load-bearing synovial joint exposed to complex mechanical demands. Its fibrocartilage and subchondral bone remodel in response to physiological loading, whereas excessive, repetitive or abnormally distributed forces may exceed the adaptive capacity of the joint and contribute to degenerative change [1,2]. Temporomandibular joint osteoarthritis (TMJOA) involves progressive deterioration of articular cartilage, subchondral bone and synovial tissues and may be accompanied by pain, restricted mandibular function and structural impairment. Its reported frequency varies substantially with population, diagnostic criteria and imaging method, and clinical findings may not reflect the extent of structural involvement [3].

Because dental occlusion contributes to the mechanical environment of both TMJs, alterations in occlusal contacts, vertical dimension, or mandibular position may modify the forces transmitted to joint tissues [2]. Experimental manipulation of these factors has therefore been used to investigate mechanically induced TMJ degeneration [2,4]. Disordered occlusion has been associated with early subchondral bone loss followed by structurally incomplete repair [5], while unilateral anterior crossbite can induce progressive chondrocyte disorganisation, proteoglycan depletion, collagen disruption and abnormal cartilage mineralisation [6]. Unilateral functional mandibular displacement may also produce osteoarthritis-like changes in both TMJs, indicating that a unilateral disturbance can generate a bilateral, side-specific response [7]. The persistence and reversibility of these changes appear to depend partly on the duration of the mechanical stimulus [8].

Magnetic resonance imaging (MRI) enables non-invasive assessment of the articular disc, joint fluid, bone marrow and other TMJ abnormalities [9], whereas histology directly characterises tissue architecture and cellular change. Agreement between MRI and histopathological grading has been reported for corresponding subchondral bone changes in advanced human TMJOA [10]. However, controlled experimental studies of occlusally induced degeneration have usually relied on histology, micro-computed tomography or molecular analyses [4,5,6,7,8], and evidence integrating serial MRI with bilateral endpoint tissue assessment remains limited.

The aim of this study was therefore to determine whether a sustained unilateral posterior occlusal alteration produces bilateral structural changes in rat TMJs and to characterise their temporal MRI and endpoint histological patterns. We hypothesised that the intervention would produce bilateral MRI abnormalities accompanied by a more pronounced tissue response in the ipsilateral joint.

## 2. Materials and Methods

### 2.1. Animals, Study Design, and Ethical Considerations

Twenty-eight female Wistar rats, aged 8 weeks and weighing approximately 250 g at the start of the experiment, were obtained from the Animal Facility of the University of Oviedo. The animals were supplied by Charles River at 6 weeks of age and acclimatised for 2 weeks. They were housed under identical lighting conditions with food and water available ad libitum. Animals were allocated by the investigator to an experimental group (n = 12), a baseline control group (n = 4) and an age-matched control group (n = 12). Group allocation was performed before the start of the experiment without applying predefined selection criteria based on body weight or other baseline characteristics. All animals were of the same strain, sex and age at the time of allocation. Experimental and age-matched control animals underwent further MRI at 16 and 24 weeks of age and were euthanised after the final examination. Female rats were used because animals of this sex, strain and age were those available from the institutional animal facility when the experiment was initiated; sex was not selected on the basis of a specific biological hypothesis. The baseline control group provided a reference for TMJ morphology at the beginning of the experimental period, whereas the 24-week age-matched control group was included to distinguish intervention-related changes from those associated with normal growth and maturation during the 16-week follow-up.

The Animal Facility of the University of Oviedo was registered as a breeding and user centre for experimental animals (registration no. 33443591) in accordance with Spanish Royal Decree 53/2013. Housing complied with Law 13/2002 of the Principality of Asturias on animal welfare and the University of Oviedo Research Ethics Committee regulations approved on 25 June 2019.

### 2.2. Induction and Monitoring of the Unilateral Occlusal Alteration

At 8 weeks of age, experimental animals were anaesthetised with inhaled isoflurane (4% for induction and 2% for maintenance). The occlusal surfaces of the three right maxillary molars were etched with 37% orthophosphoric acid (Octacid), rinsed and dried. A light-cured adhesive (Prime&Bond, Dentsply DeTrey GmbH, Konstanz, Germany) and a posterior composite resin Grandio^®^ (VOCO GmbH, Cuxhaven, Germany). were applied. The occlusal pits were filled, and the composite was shaped to create a flat occlusal surface. The alteration was maintained for 16 weeks. The amount of composite resin was not standardised by volume or weight; sufficient material was applied in each animal to obtain the intended occlusal alteration. After application and shaping of the composite resin, uniform occlusal contact across the three treated right maxillary molars was verified in vivo using articulating paper.

Animals were examined weekly to monitor body weight, composite integrity and incisor growth. Worn or fractured composite was replaced, and overgrown incisors were filed when required.

### 2.3. Magnetic Resonance Imaging

MRI examinations were performed at the Preclinical Imaging Laboratory of the Animal Facility (Unidad de Bioterio) of the University of Oviedo and Associated Healthcare Institutions. Animals were anaesthetised with isoflurane in oxygen (4% for induction and 2% for maintenance) and positioned prone. Both TMJs were examined using a 3-T benchtop system (MR Solution, Guildford, UK) and a dedicated rat-head coil. Eight axial slices were acquired for each TMJ using T1- and T2-weighted sequences (slice thickness, 0.7 mm; interslice gap, 0.1 mm). T1-weighted parameters were repetition time/echo time, 720/11; eight averages; echo-train length, 4; echo spacing, 11; 248 views; and acquisition time, 6 min 3 s. T2-weighted parameters were repetition time/echo time, 3800/68; three averages; echo-train length, 8; echo spacing, 17; 240 views; and acquisition time, 5 min 53 s. Experimental and age-matched control animals underwent bilateral TMJ MRI at 16 and 24 weeks of age, corresponding to 8 and 16 weeks after induction of the unilateral occlusal alteration in the experimental group. MRI assessment was performed by S.C., Professor of Radiology at the University of Oviedo and head of the Preclinical Imaging Laboratory.

The following features were evaluated separately in the right and left TMJs: joint-space narrowing, joint-line irregularity and condylar bone-marrow oedema (present, absent or not assessable); condylar contour alteration and joint effusion (present or absent); type of condylar contour alteration (flattening, bulbous morphology, other or none); and articular disc morphology or size (no alteration, enlarged, reduced or wrinkled). Findings were dichotomised as normal or abnormal for longitudinal analysis.

### 2.4. Tissue Collection and Histological Processing

Animals were euthanised by carbon dioxide inhalation. The heads were isolated, and soft tissues, parts of the cranial bones and the brain were removed. Specimens were decalcified for approximately 7–10 days in 10% formalin, 15.4 M nitric acid and distilled water (10:5:85, *v*/*v*/*v*), washed for 12 h and processed for paraffin embedding. Serial coronal sections were cut at 10 μm, mounted on gelatin-coated slides and dried at 45 °C for 24 h. Sections were stained with haematoxylin-eosin and Masson’s trichrome. Stained sections were used to assess the organisation and morphology of the condylar and glenoid cartilages and the articular disc and to obtain histomorphometric measurements.

### 2.5. Histological and Histomorphometric Assessment

Qualitative assessment considered cartilage stratification, extracellular matrix staining, chondrocyte morphology and distribution, and disc organisation. Five sampling segments of the central load-bearing region were evaluated in the articular disc and cartilages. Thickness was measured in the condylar cartilage, glenoid cartilage and articular disc. Disc thickness was assessed in medial, central and lateral regions by two independent observers. Histomorphometric measurements were independently performed by two investigators (S.d.C. and J.A.V.), both blinded to group allocation.

Cell density was determined by manually counting nuclei within a delineated area and was expressed as cells/mm^2^. Counts were obtained in the articular disc and in the superficial region of the articular cartilage, defined by the morphological boundary between the superficial and proliferative layers and the corresponding matrix characteristics. Measurements were performed using Olympus cellSens Dimension image-analysis software. Cell-density counts were also independently performed by S.d.C. and J.A.V. under blinded conditions. Interobserver agreement was assessed by direct comparison of the independently obtained measurements; no intraclass correlation coefficient was calculated. When the discrepancy between observers exceeded 10%, the assessment was repeated.

### 2.6. Statistical Analysis

Statistical analyses were performed with GraphPad Prism version 7.0 (GraphPad Software, La Jolla, CA, USA). Normality of continuous variables was assessed using the Kolmogorov–Smirnov test. Student’s *t*-tests were used for comparisons between two means, and paired categorical MRI variables were analysed with the McNemar test after dichotomisation as normal or abnormal. Tests were two-sided, and *p* < 0.05 was considered statistically significant.

## 3. Results

### 3.1. Magnetic Resonance Imaging Findings

The right TMJ was defined as ipsilateral to the occlusal alteration and the left TMJ as contralateral. Joint-line irregularity and condylar bone-marrow oedema were not assessable at 16 or 24 weeks. Joint-space narrowing was the earliest and most frequent MRI finding. Eight weeks after induction of the alteration (age 16 weeks), it was present in 10 of 12 right TMJs (83.3%) and 9 of 12 left TMJs (75.0%) in the experimental group; 9 animals (75.0%) showed bilateral narrowing. No narrowing was detected in age-matched controls. At 24 weeks, 11 of 12 experimental animals (91.7%) showed bilateral narrowing. Narrowing was detected in 2 of 12 right control TMJs (16.7%) and in no left control TMJs. Because the experimental animals did not undergo pre-intervention MRI, direct within-animal comparison with an 8-week baseline was not possible. Between 16 and 24 weeks, joint-space narrowing remained frequent and increased descriptively in the experimental group (Table 1).

**Figure 1:**
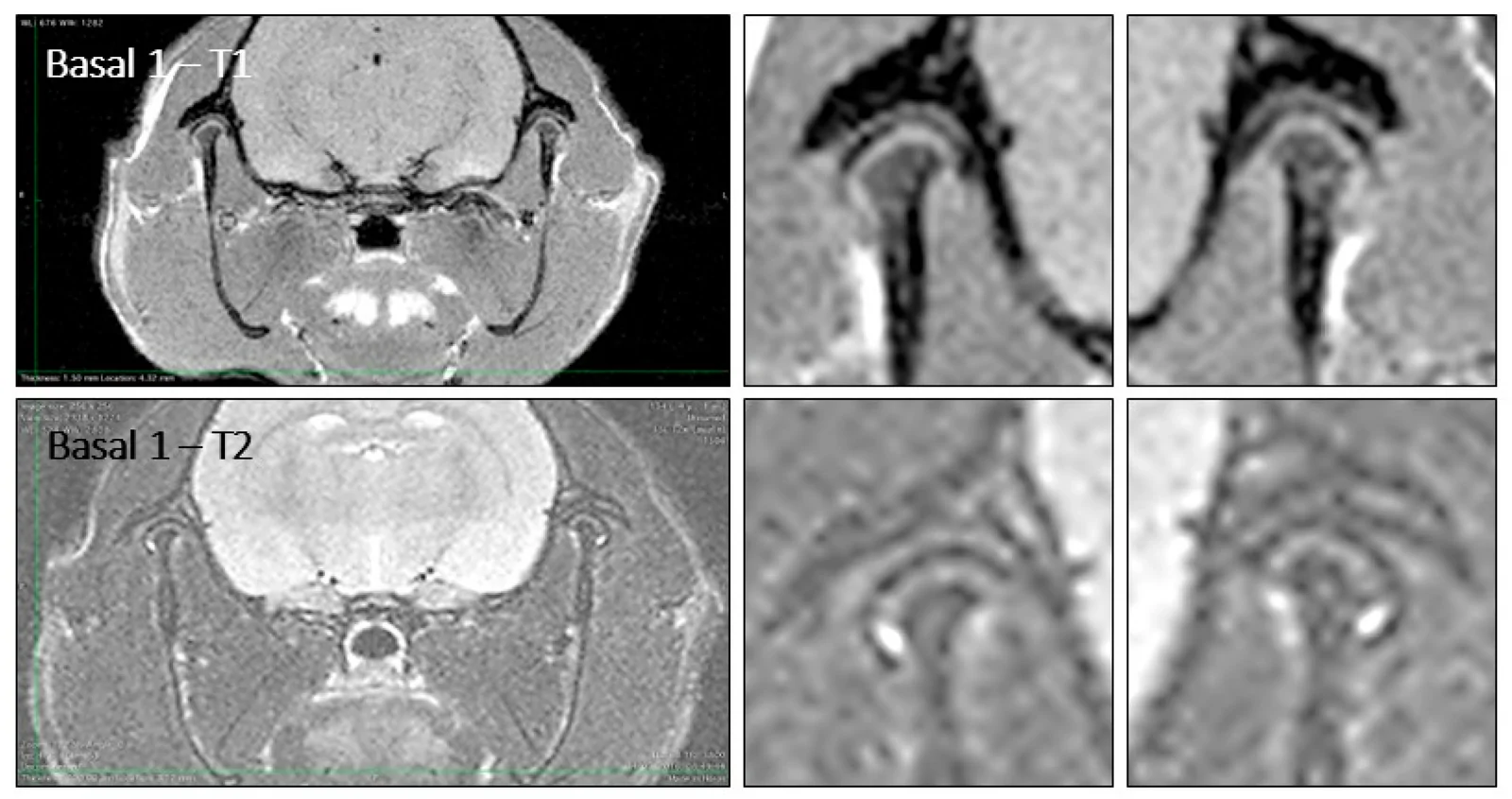
Representative T1- and T2-weighted MRI images acquired at baseline. Whole-head axial images and enlarged views of both temporomandibular joints are shown.

**Figure 2:**
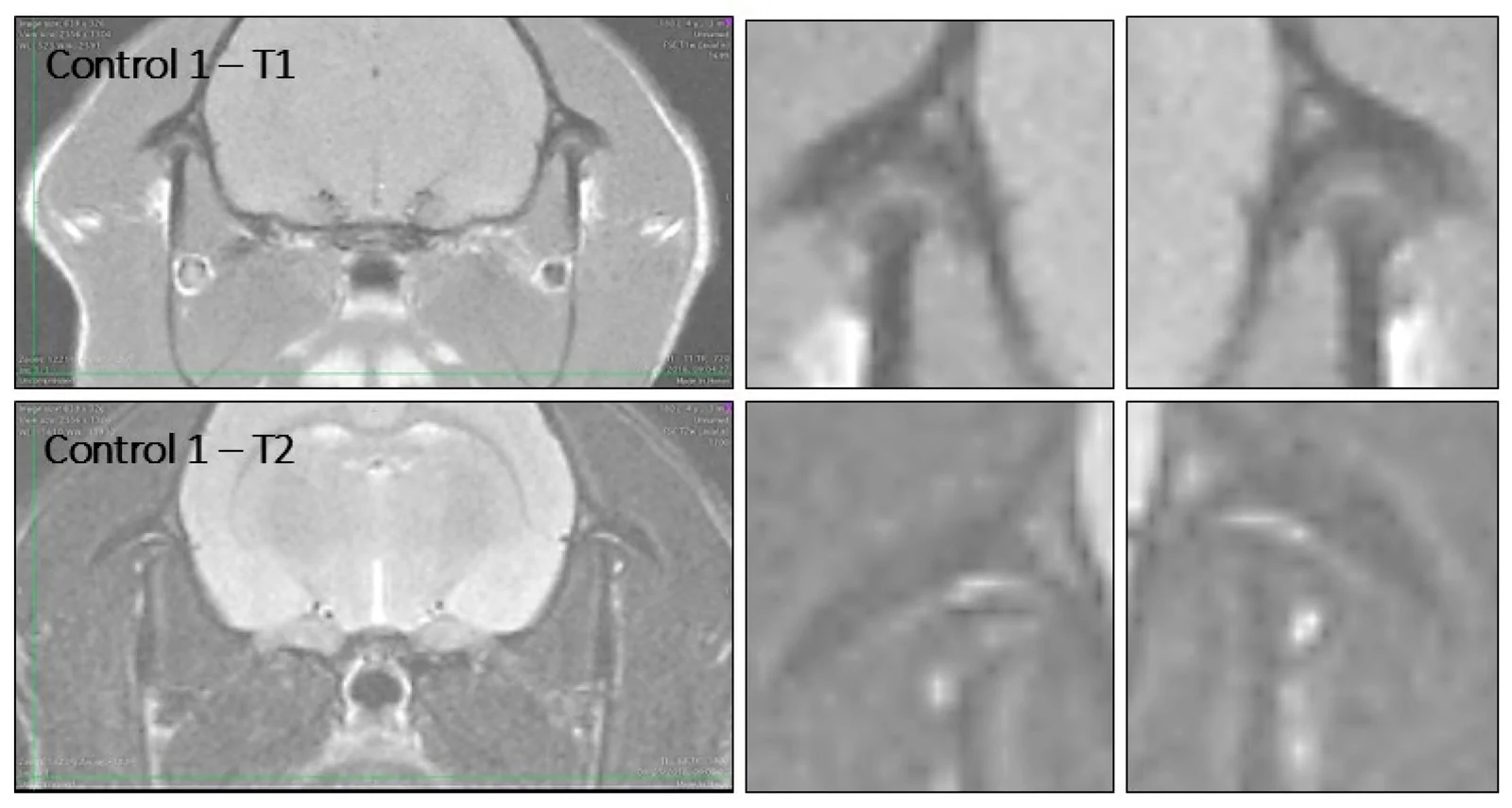
Representative T1- and T2-weighted MRI images from an animal in control group 1. Whole-head axial images and enlarged views of both temporomandibular joints are shown.

**Figure 3:**
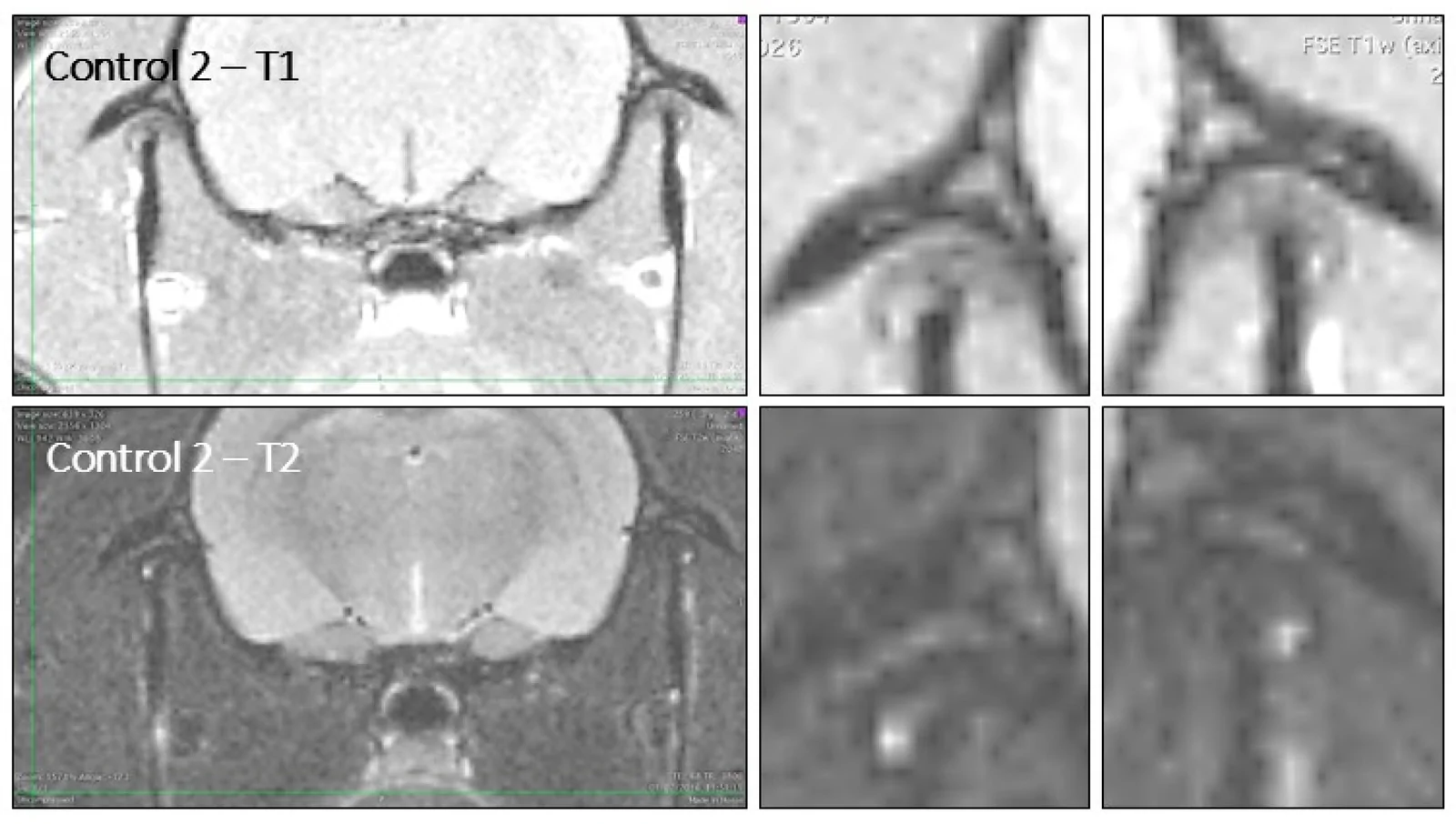
Representative T1- and T2-weighted MRI images from an animal in control group 2. Whole-head axial images and enlarged views of both temporomandibular joints are shown.

Representative T1- and T2-weighted MRI appearances at baseline and in the control groups are shown in Figure 1, Figure 2 and Figure 3.

Bilateral effusion was observed in one experimental animal at 16 weeks. At 24 weeks, effusion was detected in two experimental animals, affecting one right and one left TMJ. No effusion was detected in controls.

No control animal showed a condylar contour alteration. At 16 weeks, flattening affected 6 of 12 right TMJs (50.0%) and 4 of 12 left TMJs (33.3%) in the experimental group. One additional right and one additional left TMJ from different animals were classified as having another contour alteration. At 24 weeks, flattening affected 9 of 12 TMJs (75.0%) on each side, and one additional right condyle had a bulbous alteration. The prevalence of condylar contour alteration increased between 16 and 24 weeks descriptively on both sides.

Disc morphology also changed progressively. At 16 weeks, 5 of 12 experimental TMJs (41.7%) on each side had no disc alteration, 5 (41.7%) had reduced disc size, and 2 (16.7%) had a wrinkled appearance. All right control discs were classified as normal; one left control disc was reduced. At 24 weeks, reduced disc size was recorded in all experimental TMJs and in 3 of 12 control TMJs (25.0%) on each side. Because pre-intervention MRI was not available for the experimental animals, no direct intra-individual baseline comparison was possible; disc abnormalities nevertheless progressed between 16 and 24 weeks descriptively.

### 3.2. Structural Histological Findings

In baseline and age-matched controls, the condylar and glenoid cartilages retained the expected five-layer organisation: superficial or fibrous, proliferative, intermediate, hypertrophic and calcified layers. Superficial chondrocytes had normal morphology and were oriented parallel to the articular surface. The articular discs showed regular connective tissue and fibrocartilage organisation, with large, rounded nuclei aligned parallel to the articular surface. No side-to-side structural differences were evident.

At 24 weeks, structural changes were concentrated in the ipsilateral TMJ. The condylar and glenoid cartilages were thickened, and in most experimental animals, the extracellular matrix had lost its characteristic mixed magenta-blue staining and normal stratification was no longer distinguishable. Chondrocytes appeared flattened, with large nuclei and irregular distribution. The ipsilateral disc was also thickened and showed abnormal nuclear orientation, although its laminar organisation remained identifiable. Contralateral cartilage and disc retained an architecture comparable to that of age-matched controls.

**Figure 4:**
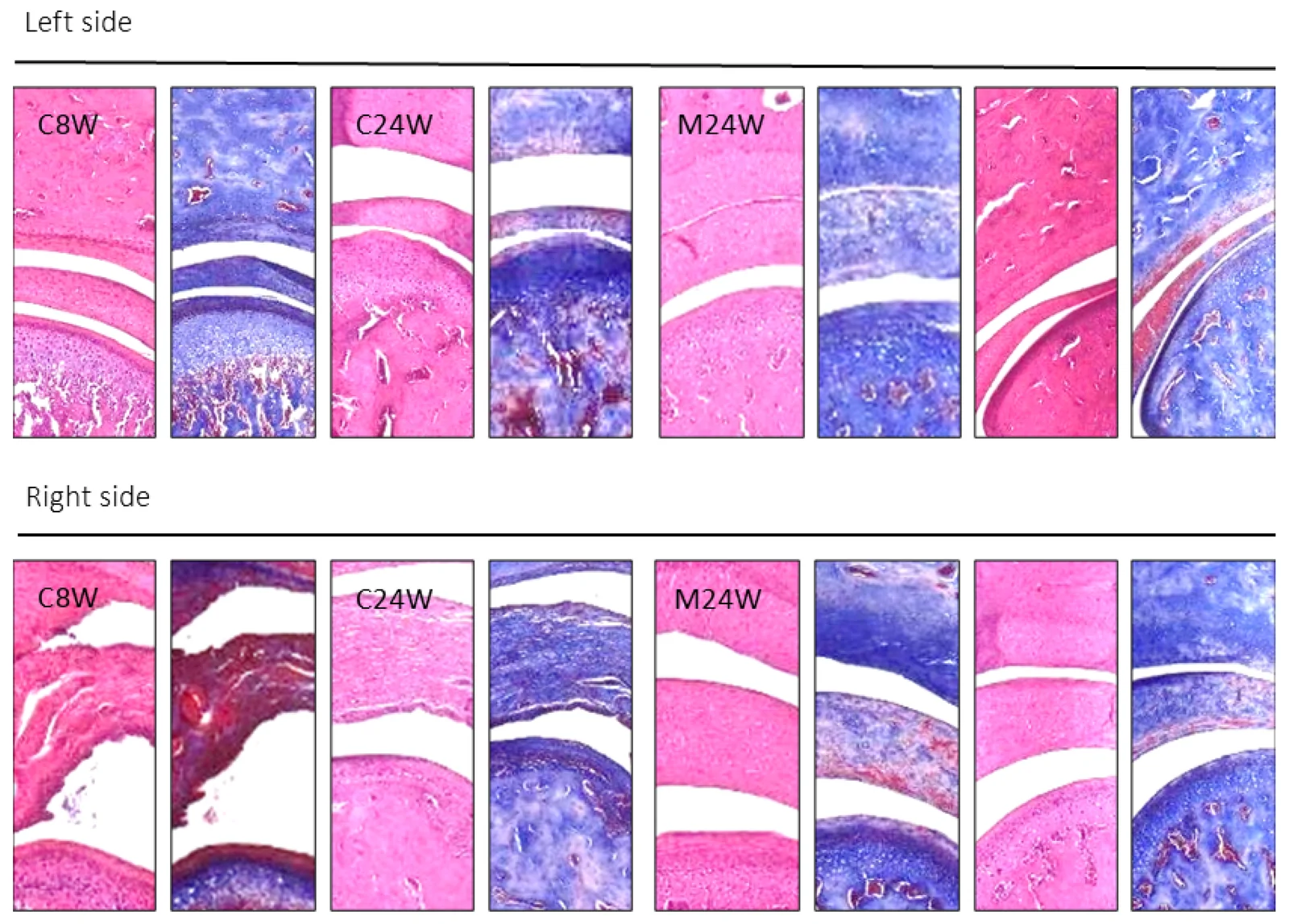
Representative coronal histological sections of the left and right temporomandibular joints from control and experimental animals. Haematoxylin–eosin and Masson’s trichrome staining are shown; group and time-point labels are indicated in the image.

Representative histological micrographs are shown in Figure 4.

### 3.3. Histomorphometric Findings

The group-level values reported for tissue thickness and cell density are summarised in Table 2. Compared with baseline controls at 8 weeks, 24-week controls had lower condylar cartilage thickness on the right (182 versus 161 μm) and left (188 versus 169 μm; *p* < 0.05). Glenoid cartilage and disc thickness did not differ significantly between the two control ages.

Intervention-related thickness differences were greatest ipsilaterally. At 24 weeks, ipsilateral condylar cartilage thickness was 318 μm, compared with 161 μm in the corresponding age-matched control TMJ and 186 μm in the contralateral experimental TMJ (*p* < 0.01 for both comparisons). Ipsilateral glenoid cartilage thickness was 203 μm, compared with 160 μm in age-matched controls and 181 μm contralaterally (*p* < 0.05 for both). Ipsilateral disc thickness was 406 μm, compared with 189 μm in age-matched controls (*p* < 0.01) and 302 μm contralaterally (*p* < 0.05). The contralateral disc was also thicker than the corresponding age-matched control disc (302 versus 201 μm).

Cell-density changes showed the same asymmetric pattern. Compared with baseline controls at 8 weeks, condylar cartilage cell density was lower in 24-week controls (right, 228 versus 182 cells/mm^2^; left, 217 versus 189 cells/mm^2^; *p* < 0.05), whereas glenoid cartilage and disc cell density did not differ significantly between control ages. At 24 weeks, cell density was lower ipsilaterally than in the corresponding age-matched control and contralateral experimental TMJs in glenoid cartilage (138 versus 189 and 174 cells/mm^2^, respectively; *p* < 0.05), condylar cartilage (122 versus 182 and 194 cells/mm^2^; *p* < 0.01 and *p* < 0.05) and articular disc (134 versus 187 and 167 cells/mm^2^; *p* < 0.01 and *p* < 0.05).

## 4. Discussion

This study revealed a bilateral but asymmetric TMJ response to a unilateral posterior occlusal alteration. By 24 weeks, MRI showed bilateral joint-space narrowing in 11 of 12 animals, condylar flattening in 9 of 12 TMJs on each side and reduced disc size in every experimental joint. Tissue analysis refined this pattern: cartilage and disc thickening, loss of normal organisation and reduced cellularity were concentrated ipsilaterally, while contralateral architecture remained preserved and disc thickness showed a smaller quantitative change. This multimodal pattern is the central finding of the study and shows that a unilateral occlusal disturbance can affect both TMJs, but with different expression and intensity in each joint.

The TMJ response to mechanical stimulation spans adaptation, maladaptive remodelling and progressive degeneration according to the magnitude, direction and duration of loading, as well as age and the tissue examined [11]. In the closest published model, Wang et al. [4] increased the occlusal vertical dimension in adult rats using a standardised bilateral occlusal plate and observed early condylar cartilage damage followed by repair-like changes at 8 weeks; damage developed faster and was more severe than after occlusal loss. The present findings extend this time-dependent response to a unilateral posterior alteration and show that its effects are expressed across both joints.

The longitudinal MRI data define an early and progressively more extensive bilateral response. Joint-space narrowing was already frequent 8 weeks after intervention, while condylar flattening and reduced disc size became more prevalent by the endpoint. The simultaneous progression of joint-space, condylar, and disc abnormalities identifies a coordinated structural remodelling phenotype rather than an isolated imaging change.

The bilateral distribution is consistent with previous unilateral functional models. Wattanachai et al. [12] found opposite extracellular-matrix responses after a unilateral functional mandibular shift: type II collagen and aggrecan increased transiently contralaterally but decreased throughout observation ipsilaterally. Zou et al. [7] likewise reported progressively aggravated osteoarthritis-like changes in both condyles after experimental lateral mandibular displacement. Although the appliances and loading patterns differed, these studies and the present results converge on the same principle: a unilateral disturbance can modify both TMJs while generating side-specific responses. Redistribution of mandibular loading across the coupled joints provides a coherent explanation for bilateral MRI change alongside the stronger ipsilateral tissue response.

The difference between bilateral MRI abnormalities and the asymmetric histological pattern is therefore informative. MRI followed whole-joint features over time, including joint-space width, overall condylar contour and global disc morphology, whereas histology localised endpoint changes in tissue architecture, thickness and cellularity. Li et al. [10] reported substantial MRI–histopathology agreement for graded subchondral bone changes in human TMJOA, supporting the value of combining structural imaging with tissue assessment. MRI is particularly suited to serial evaluation of the disc, effusion and other soft-tissue changes, while histology provides cellular and matrix detail [13]. In the present model, the two methods captured complementary levels of the same response: bilateral progression at the whole-joint level and predominantly ipsilateral remodelling at the tissue level.

Ipsilateral condylar and glenoid cartilages were thicker, had lost their normal layered organisation and staining pattern and contained flattened, irregularly distributed cells; cell density was also lower in ipsilateral cartilage and disc. The biological meaning of increased thickness depends on its accompanying tissue features. Utreja et al. [14] reported thicker condylar cartilage after 5 days of low-magnitude static loading together with increased cell proliferation and matrix formation, a pattern of adaptive remodelling. By contrast, Xie et al. [15] found that cartilage thickening at 4 weeks coexisted with cellular disorganisation, local cell loss and surface fibrillation before thinning and more severe damage developed at 8 and 12 weeks. In the present study, the combination of increased thickness, reduced cellularity, altered matrix staining and loss of stratification identifies maladaptive remodelling with early degenerative-like features. The cartilage thinning described in other models [4,7] further indicates that tissue thickness reflects the stage and type of loading response rather than severity in isolation.

The articular disc showed the largest histological increase in thickness ipsilaterally, accompanied by abnormal nuclear orientation and reduced cell density despite preserved laminar organisation. Sun et al. [16] similarly observed selective thickening of the intermediate zone after experimentally induced non-balanced occlusion and interpreted it as adaptation to an altered condyle–fossa relationship. Their intervention differed from the present posterior alteration, but both studies identify the disc as a responsive component of occlusally induced TMJ remodelling. Reduced disc size on MRI and increased local thickness on histology describe different dimensions in different planes; considered together, they provide convergent evidence of disc remodelling.

Age-matched controls clarified the contribution of maturation. Condylar cartilage thickness and cell density were lower at 24 weeks than in baseline controls at 8 weeks, a direction of change also documented by Chen et al. [17] in mouse mandibular condylar cartilage. Against this maturational background, the much larger ipsilateral thickness changes, reduced cellularity and altered tissue organisation define an intervention-related response beyond normal age-associated change. Recent experimental evidence using unbalanced occlusal loading likewise demonstrates side-dependent remodelling of condylar cartilage, subchondral bone and the articular disc, further supporting the sensitivity of the TMJ to altered occlusal mechanics [18].

The exclusive use of female animals also deserves consideration in the biological interpretation of the present findings. Temporomandibular disorders are more prevalent in women than in men, and imaging studies have also reported a higher prevalence and severity of temporomandibular joint osteoarthritis in women [19,20]. Thus, although female rats were originally used because of animal availability rather than to investigate a sex-specific hypothesis, the present model addresses a clinically relevant population characteristic. Nevertheless, because males were not included, the present study cannot determine whether the magnitude or pattern of TMJ remodelling following unilateral occlusal alteration is sex-dependent.

Taken together, the longitudinal bilateral MRI design and endpoint assessment of condylar cartilage, glenoid cartilage and articular disc establish a coherent temporal–spatial sequence. Imaging abnormalities emerged within 8 weeks and progressed bilaterally; by 24 weeks, tissue remodelling was most pronounced ipsilaterally, with subtler contralateral quantitative changes. This resolution across time, side and tissue is the main strength of the study and supports the model as a platform for investigating early occlusion-related TMJ remodelling.

This study has some limitations. First, the sample size was limited, although it was sufficient to demonstrate consistent longitudinal MRI and histological changes across the experimental groups. Second, functional outcomes such as masticatory performance or pain-related behaviour were not assessed, preventing direct correlation between structural alterations and functional impairment. Finally, this experimental model was designed to investigate the biological consequences of a sustained unilateral posterior occlusal alteration in rats and, therefore, caution is warranted when extrapolating these findings to human temporomandibular joint disorders. Nevertheless, the combined longitudinal MRI and endpoint histological assessment provides complementary evidence of bilateral but asymmetric joint remodelling and represents one of the main strengths of the present study. No formal a priori sample-size calculation was performed, and no additional animals were included specifically to compensate for potential attrition; no animals were lost during follow-up. Future studies should determine sample size prospectively using a predefined primary outcome, expected effect size and variability, desired statistical power, and anticipated animal loss. Experimental animals did not undergo pre-intervention MRI; baseline imaging was obtained from a separate 8-week control group. Consequently, direct intra-individual MRI comparison between the pre-intervention state and subsequent time points was not possible. Because only female rats were included, potential sex-related differences in TMJ adaptation and remodelling could not be assessed, and the findings should not be extrapolated to males without confirmation. Individual vitamin C and vitamin D status was not assessed because micronutrient metabolism was outside the scope of the study; although all animals received the same standard diet under identical housing conditions, a potential contribution of individual micronutrient status to variability in TMJ tissue responses cannot be formally excluded.

## 5. Conclusions

Unilateral posterior occlusal alteration produced a progressive bilateral but asymmetric TMJ response in rats. MRI documented bilateral joint-space narrowing, condylar flattening and reduced disc size, while histology and histomorphometry showed cartilage and disc thickening, loss of normal tissue organisation and reduced cellularity predominantly in the ipsilateral joint, with subtler contralateral disc changes. Together, these findings show that a unilateral occlusal disturbance can affect both TMJs to different degrees and support this model for investigating early load-related degenerative remodelling.

## Figures and Tables

**Table 1:** Bilateral MRI findings at 16 and 24 weeks of age.

Outcome	Age	Control Right	Experimental Right	Control Left	Experimental Left
Joint-space narrowing	16 wk	0/12 (0%)	10/12 (83.3%)	0/12 (0%)	9/12 (75.0%)
Joint-space narrowing	24 wk	2/12 (16.7%)	11/12 (91.7%)	0/12 (0%)	11/12 (91.7%)
Joint effusion	16 wk	0/12 (0%)	1/12 (8.3%)	0/12 (0%)	1/12 (8.3%)
Joint effusion	24 wk	0/12 (0%)	1/12 (8.3%)	0/12 (0%)	1/12 (8.3%)
Condylar flattening	16 wk	0/12 (0%)	6/12 (50.0%)	0/12 (0%)	4/12 (33.3%)
Condylar flattening	24 wk	0/12 (0%)	9/12 (75.0%)	0/12 (0%)	9/12 (75.0%)
Reduced disc size	16 wk	0/12 (0%)	5/12 (41.7%)	1/12 (8.3%)	5/12 (41.7%)
Reduced disc size	24 wk	3/12 (25.0%)	12/12 (100%)	3/12 (25.0%)	12/12 (100%)

Values are n/N (%). At 16 weeks, one additional experimental TMJ on each side had another contour alteration and two discs on each side were wrinkled. At 24 weeks, one additional right condyle was bulbous.

**Table 2:** Histomorphometric outcomes at baseline and 24 weeks.

Outcome	Baseline Control R	Baseline Control L	24-Week Control R	24-Week Control L	24-Week EI (R)	24-Week EC (L)
Condylar cartilage thickness, μm	182.6 ± 6.3	188.7 ± 6.8	161.1 ± 7.7	169.3 ± 5.6	318.4 ± 8.2	186 ± 8.1
Glenoid cartilage thickness, μm	166.1 ± 6.1	171.6 ± 4.9	160.8 ± 4.7	151.3 ± 5.0	203 ± 4.3	181.1 ± 4.4
Articular disc thickness, μm	215.3 ± 5.7	221.0 ± 3.9	189.6 ± 5.2	201.3 ± 4.1	406.5 ± 6.8	302.3 ± 7.2
Glenoid cartilage cell density, cells/mm^2^	215.9 ± 8.9	221.2 ± 8.3	189.2 ± 3.3	201.2 ± 6.7	138.1 ± 4.1	174.3 ± 3.6
Condylar cartilage cell density, cells/mm^2^	228.3 ± 5.8	217.1 ± 6.1	182.6 ± 4.3	189.7 ± 5.6	122.7 ± 4.8	194.5 ± 5.1
Articular disc cell density, cells/mm^2^	204.3 ± 4.3	214.6 ± 7.2	187.8 ± 5.3	194.3 ± 5.5	134.8 ± 4.9	167.0 ± 4.6

R, right; L, left; EI, experimental ipsilateral; EC, experimental contralateral. Values are presented as mean ± SD; n = 14.

## Data Availability

The datasets generated and analysed during the current study are available from the corresponding author upon reasonable request.
